# Genetic Susceptibility to Acute Rheumatic Fever: A Systematic Review and Meta-Analysis of Twin Studies

**DOI:** 10.1371/journal.pone.0025326

**Published:** 2011-09-30

**Authors:** Mark E. Engel, Raphaella Stander, Jonathan Vogel, Adebowale A. Adeyemo, Bongani M. Mayosi

**Affiliations:** 1 Department of Medicine, Groote Schuur Hospital and University of Cape Town, Cape Town, South Africa; 2 Center for Research in Genomics and Global Health, National Human Genome Research Institute, National Institutes of Health, Bethesda, Maryland, United States of America; University of Hong Kong, Hong Kong

## Abstract

**Background:**

Acute rheumatic fever is considered to be a heritable condition, but the magnitude of the genetic effect is unknown. The objective of this study was to conduct a systematic review and meta-analysis of twin studies of concordance of acute rheumatic fever in order to derive quantitative estimates of the size of the genetic effect.

**Methods:**

We searched PubMed/MEDLINE, ISI Web of Science, EMBASE, and Google Scholar from their inception to 31 January 2011, and bibliographies of retrieved articles, for twin studies of the concordance for acute rheumatic fever or rheumatic heart disease in monozygotic versus dizygotic twins that used accepted diagnostic criteria for acute rheumatic fever and zygosity without age, gender or language restrictions. Twin similarity was measured by probandwise concordance rate and odds ratio (OR), and aggregate probandwise concordance risk was calculated by combining raw data from each study. ORs from separate studies were combined by random-effects meta-analysis to evaluate association between zygosity status and concordance. Heritability was estimated by fitting a variance components model to the data.

**Results:**

435 twin pairs from six independent studies met the inclusion criteria. The pooled probandwise concordance risk for acute rheumatic fever was 44% in monozygotic twins and 12% in dizygotic twins, and the association between zygosity and concordance was strong (OR 6.39; 95% confidence interval, 3.39 to 12.06; P<0.001), with no significant study heterogeneity (P = 0.768). The estimated heritability across all the studies was 60%.

**Conclusions:**

Acute rheumatic fever is an autoimmune disorder with a high heritability. The discovery of all genetic susceptibility loci through whole genome scanning may provide a clinically useful genetic risk prediction tool for acute rheumatic fever and its sequel, rheumatic heart disease.

## Introduction

Acute rheumatic fever is a multifactorial disorder that is caused by an interaction between a rheumatogenic strain of group A streptococcus and a susceptible host who lives in poor social conditions. The role of a host susceptibility factor that may be common to all human beings is supported by the observation that the lifetime cumulative incidence of acute rheumatic fever in populations who are exposed to untreated group A streptococcal pharyngitis is 3–6% regardless of geography or ethnicity. [1] While much is known about the social factors and the microbial agent that predispose to acute rheumatic fever, little progress has been made in elucidating genetic susceptibility factors that are reproducible in different populations. [1]

Several twin and family aggregation studies have suggested a genetic effect, but they have not provided a quantitative estimate of the magnitude of the genetic contribution in acute rheumatic fever. Furthermore, the molecular genetic studies of human leukocyte antigen (HLA) and non-HLA factors have been characterized by small studies with inconsistent and conflicting findings. [1] A quantitative assessment of the genetic effect in twin or adoption studies, which provide a reliable estimate of genetic effect in familial conditions, will provide guidance on the desirability of embarking upon the expensive ‘global genome analysis’ studies of the condition that have been recommended recently. [1] Higher concordance rates between monozygotic twins compared with dizygotic twins indicate a greater role for genetic factors in the development of a disease given that monozygotic twins are genetically identical, versus dizygotic twins who, on average, share 50% of their genes, Conversely, concordance of a similar magnitude in both monozygotic and dizygotic twin pairs suggests the involvement of factors not pertaining to genes. [2] It would thus be prudent to invest scarce resources on genetic studies of conditions with good evidence of genetic determination particularly in resource-poor countries where acute rheumatic fever is prevalent.

We have conducted a systematic review and meta-analysis of the concordance rate and heritability of acute rheumatic fever in monozygotic and dizygotic twin pairs as reported in observational studies. The primary aim was to determine the extent to which the variation of the disease between monozygotic and dizygotic twin pairs is due to genetic effects.

## Methods

The protocol for this review, which was not registered, is available from the authors upon request.

### Study selection and characteristics

We included reports which met the following criteria: 1) twin studies reporting on the concordance for acute rheumatic fever and/or rheumatic heart disease in monozygotic versus dizygotic twins (i.e., comparative group used); 2) use of accepted diagnostic criteria for acute rheumatic fever; 3) clear indication of how zygosity was established; and 4) diagnostic assessment of relatives preferably performed with investigators blind to the affection status of the proband. The first three criteria were an absolute requirement for inclusion of a report in the study. Three observers (MEE, RS, and JV) independently evaluated the titles and abstracts of search outputs, and thereafter compiled a list of articles deemed to be potentially relevant. Full-text articles were retrieved and evaluated against the inclusion criteria.

### Data sources and search strategy

Using the terms ((RHEUMATIC FEVER OR RHEUMATIC HEART) AND (FAMIL* OR TWIN OR ADOPTION)), we searched PubMed/MEDLINE, EMBASE, Thomson Reuters ISI Web of Science and Google Scholar for all reports of original research from the inception date of each database to 31 January 2011, without any language restriction. Predefined criteria were used to identify studies examining the concordance for acute rheumatic fever and rheumatic heart disease in monozygotic versus dizygotic twins. The literature search was performed independently by three of the reviewers (MEE, RS, and JV). The search strategy for Medline and EMBASE appears in Appendix S1. This process was complemented by reviewing the reference list of all articles identified, and by scanning abstracts from conference proceedings.

### Validity assessment of included studies

We assessed studies in terms of case definition and determination of twin zygosity.

### Data abstraction

From each study, two reviewers (RS, JV) independently recorded the year of publication, origin and demographic details of participants, matching procedures for zygosity, diagnostic criteria for acute rheumatic fever and rheumatic heart disease, and information on disease. A third reviewer (MEE) verified the data extraction. The remaining reviewers (AAA and BMM) served as arbitrators where necessary.

### Quantitative data synthesis

Concordance or twin similarity was assessed by calculation of two concordance rates: the pairwise concordance rate and the probandwise concordance rate. The pairwise concordance rate is given by the formula: number of pairs where both twins are affected/Total number of twins. The probandwise concordance rate is given by the formula: number of probands whose co-twins are affected/Total number of probands. The advantage of the probandwise concordance rate is that it is independent of ascertainment. We also calculated aggregate pooled probandwise concordance by combining raw data from each of the studies. Heritability was estimated by estimating the variance components using structural equation modelling techniques as implemented in the MX software package (http://www.vcu.edu/mx/). We considered an “ACE” variance components (VC) model incorporating parameters for additive genetic (A), common (shared) environmental (C), and individual specific (unshared) environmental (E) components of the total variance (V). The A, C, and E parameters were estimated by maximum likelihood, under a “liability threshold” model. Heritability (h^2^) is the proportion of the total variance that is attributable to the genetic variance; i.e. h^2^ = A/V. Odds ratios from separate studies were combined by random-effects meta-analysis according to the Mantel-Haenszel method to evaluate association between zygosity status and concordance. Heterogeneity between studies was evaluated with the *χ^2^* Q statistic which was considered significant for p<0.1. STATA software version 10 (STATA Corporation, College Station, Texas, USA) was used to perform the meta-analysis and produce the forest plots using the metan routine, which automatically adds 0·5 to all zero-containing cells of the 2×2 table before analysis. [3]

## Results

### Flow of included studies

**Figure 1:**
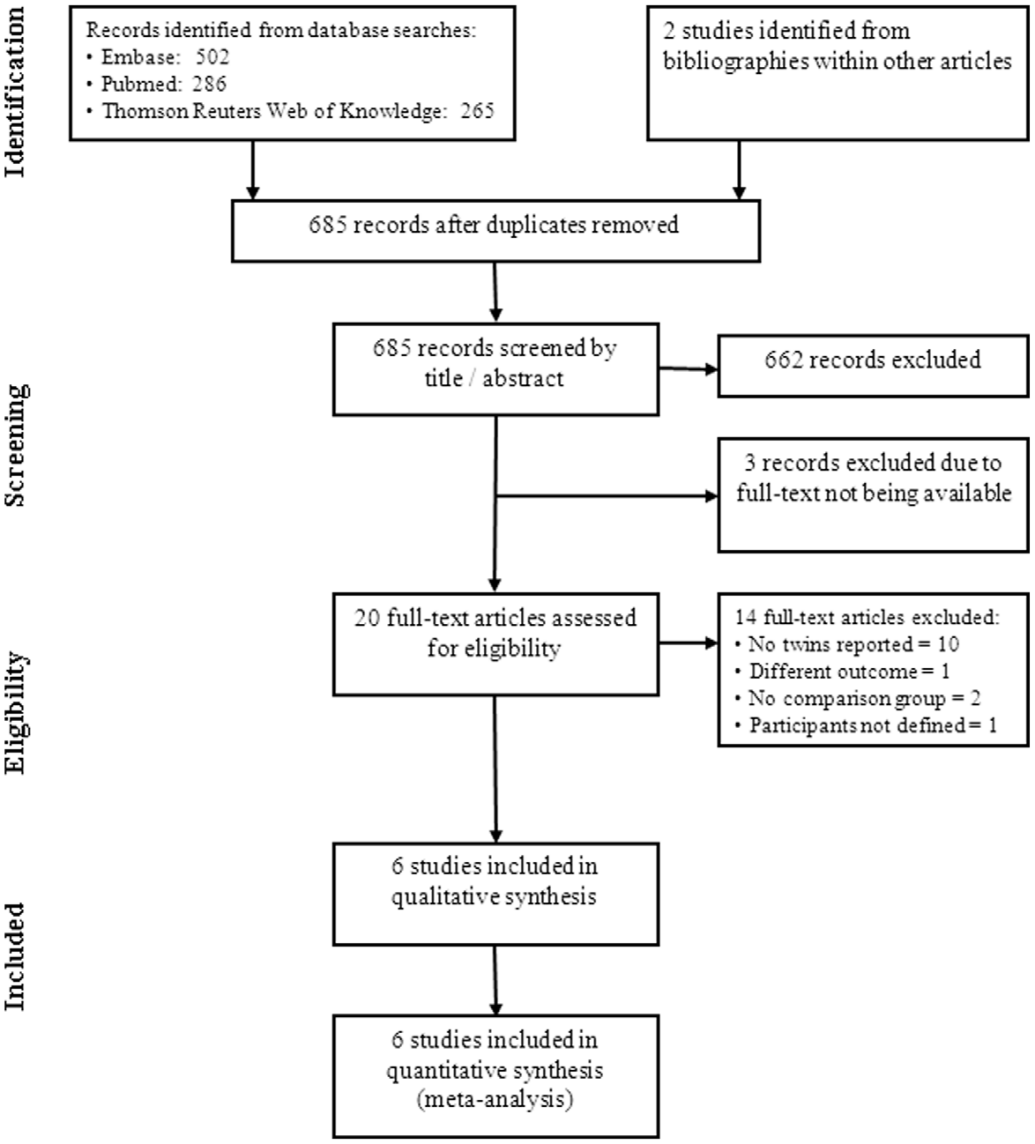
Flow chart of systematic literature search.

The electronic literature search yielded 685 articles for consideration (Figure 1). Titles and abstracts were reviewed and together with the articles identified by hand searching, Twenty-three publications were selected for possible inclusion; however, three non-English language papers were excluded due to unavailability of the full text of the articles from the authors and library archives [4], [5], [6]. Of the remaining 20 articles, a further 14 were excluded for the following reasons: no twins reported (n = 10) [7], [8], [9], [10], [11], [12], [13], [14], [15], [16], having rheumatoid arthritis as the outcome (n = 1) [17], or lack of a comparison group (n = 2). [18], [19]. Finally, one study did not distinguish between types of twins. [20] (Table 1). We did not discover any unpublished studies nor did we find any twin studies of rheumatic heart disease that met the inclusion criteria.

**Table 1:** Studies that were excluded from the systematic review.

Author	Year	Reason for exclusion
Benevolenskaia	1973	No distinction between MZ and DZ twins
Benevolenskaia	1980	No twins reported in sample
Bobylev	1972	No distinction between MZ and DZ twins
Denbow	1999	No comparison group
Dixon	1969	Different Diagnosis (Rheumatoid Arthritis)
Giannini	1967	No twins reported in sample
Honeyman	1971	No distinction between MZ and DZ twins
Horne	2004	No twins reported in sample
Paul(a)	1931	No twins reported in sample
Paul(b)	1931	No twins reported in sample
Perry	1940	Case study, no comparison group
Prasad	1969	No twins reported in sample
Quinn	1967	No twins reported in sample
Spagnuolo	1968	No twins reported in sample
**Not available**		
Strusberg	1968	Spanish
Uribarri	1965	Spanish
Zajicek	1966	Czech

MZ, monozygotic; DZ, dizygotic.

### Characteristics of Included twin data sets

Six studies published between 1933 and 1964 and contributing a total of 435 twin pairs were included in the analysis (Table 2). [21], [22], [23], [24], [25], [26] Two of the included datasets [21], [22] were retrieved from Perry [18] and Honeyman [20] respectively, while another was reported as a conference abstract. [26] Where indicated by the authors, studies were conducted in North America [24], [25] and Ireland. [23] One study [26] specifically focused on twins, while in the others, twin pairs formed part of larger cohorts. Wilson used previous records of children attending a pediatric cardiac clinic to obtain twin samples. [25]

**Table 2:** Observational studies of zygosity and concordance for rheumatic fever.

Study ID	No of twin pairs	Type of zygosity	Occurrence of acute rheumatic fever	
			*Concordance*	*Disconcordance*
Irvine-Jones 1933 [24]	7	MZ	2	0
		DZ	0	5
Wilson 1937 [25]	6	MZ	2	0
		DZ	2	2
Kaufmann 1938 [21]	72	MZ	5	22
		DZ	1	44
Stevenson 1953 [23]	10	MZ	1	0
		DZ	0	9
Taranta 1959 [26]	56	MZ	3	13
		DZ	1	39
Reed 1964 [22]	284	MZ	36	91
		DZ	11	146
Totals	435	MZ	49	126
		DZ	15	245

No, number; MZ, monozygotic; DZ, dizygotic.

In all six studies the outcome measured was the concordance of acute rheumatic fever in monozygotic and dizygotic twin pairs. Only two studies provided information on how zygosity was determined [23], [26]; methods included evaluation of blood groups, similarity of dermatoglyphics, hair and eye colour. Information on age and gender was incomplete or not specified: one study comprised only females in the MZ group while the DZ twin sets included both females and mixed gender. [23]. Another study consisted of mixed and same sex twin sets in a 3∶5 ratio. [26]. Where indicated, the diagnosis of acute rheumatic fever was made either using the criteria of the Heart Committee of New York Tuberculosis and Health Association, Inc or the Jones Criteria (Tables 2 and 3). [23], [24], [25], [26].

**Table 3:** Possible sources of bias: defining characteristics in individual studies.

Study ID	ARF case definition	Gender of twin pairs	Zygosity Determination
Irvine-Jones 1933 [24]	ARF, chorea, joint pain, fever absent, mitral stenosis	nd	nd
Wilson 1937 [25]	New York TB and Heart Association	nd	nd
Kaufmann 1938 [21]	nd	nd	nd
Stevenson 1953 [23]	ARF, chorea, mitral stenosis, death from RHD, rheumatic conditions with mitral involvement	MZ∶F; DZ: 6 = same sex, 3 = mixed	nd
Taranta 1959 [26]	Jones' criteria	MZ∶nd; DZ: 23 = same sex, 17 = mixed	blood group, hair and eye-colour, similarity of dermatoglyphics,
Reed 1964 [22]	nd	nd	nd

ARF, acute rheumatic fever; nd, no data; RHD, rheumatic heart disease; MZ, monozygotic; DZ, dizygotic; F, female; M, male.

### Quantitative data synthesis: relationship between zygosity and concordance rates of acute rheumatic fever

**Figure 2:**
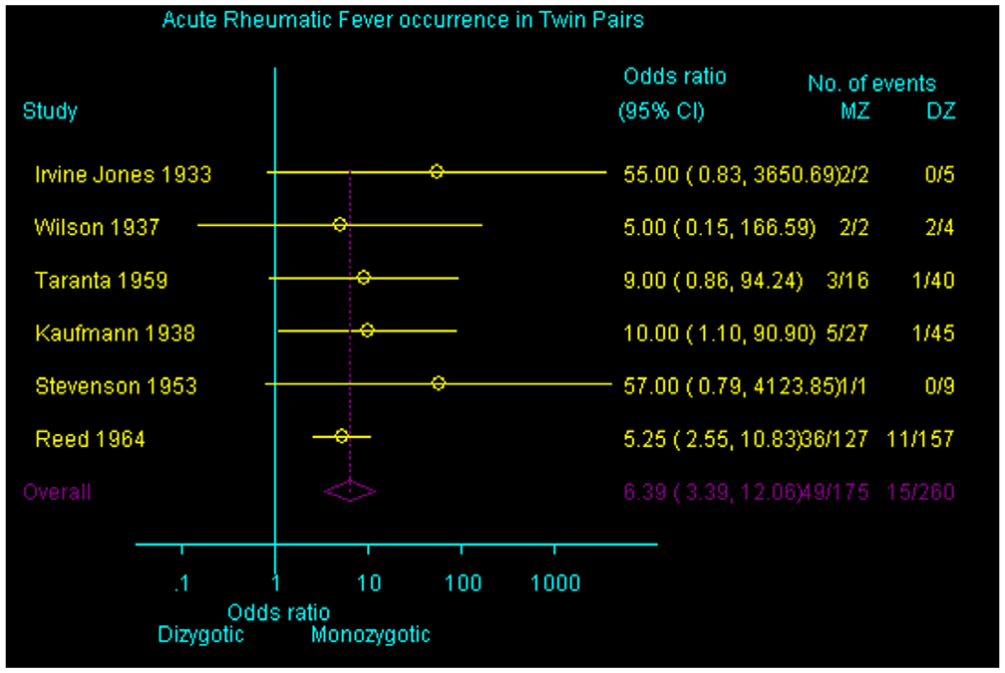
Odds ratio of concordance for acute rheumatic fever according to type of zygosity. Horizontal lines, 95% confidence interval; CI, Confidence interval; MZ, monozygotic; DZ, dizygotic.

Probandwise concordance rates ranged from 31% to 100% for monozygotic twins, and from 0% to 67% in dizygotic twins in individual studies. The pooled probandwise concordance rate was 44% for monozygotic twins and 12% for dizygotic pairs. Random-effects meta-analysis confirmed the strong association between zygosity and concordance for acute rheumatic fever in twins (OR, 6.39; 95% CI, 3.39 to 12.06; P<0.001) (Figure 2).

If the two studies with no events among dizygotic twins are excluded, the pooled OR = 5.78 (95% CI, 3.02 to 11.05; P<0.001). No statistical heterogeneity was present between studies (*χ* ^2^ = 2.56; P = 0.768). Heritability estimated by fitting VC models is shown in Table 4. Study-specific heritability estimates of acute rheumatic fever from the three larger studies varied from 0.54 to 0.73. The heritability across all the studies was 0.603 (95% confidence interval 0.413, 0.805).

**Table 4:** Results of fitting the ‘ACE’ threshold model to estimate genetic and environmental components of variance*.

Study ID	No of twin pairs	Additive genetic	Common environment	Unique environment
		*h^2^*	*c^2^*	*e^2^*
Kaufmann 1938 [21]	72	0.728	0.000	0.272
		(0.134–0.856)	(0.000–0.503)	(0.144–0.454)
Taranta 1959 [26]	56	0.714	0.018	0.268
		(0.017–0.887)	(0.000–0.565)	(0.114–0.513)
Reed 1964 [22]	284	0.540	0.275	0.185
		(0.315–0.781)	(0.051–0.474)	(0.135–0.246)
All studies**	435	0.603	0.209	0.188
		(0.413–0.805)	(0.023–0.378)	(0.144–0.239)

‘ACE’, **A**dditive genetic factors, **C**ommon environment, and unique **E**nvironment; No, number.

*Limited to studies with sufficient numbers (>50) of twin pairs.

**Includes subjects from all six studies.

The comprehensive literature search revealed no twin studies of concordance of rheumatic heart disease.

## Discussion

To the best of our knowledge, this meta-analysis of 175 monozygotic and 260 dizygotic twin pairs represents the largest quantitative assessment of the heritability of acute rheumatic fever to date. This study shows that the risk of acute rheumatic fever in a monozygotic twin with a history of acute rheumatic fever in the co-twin is increased by more than six times compared to that of dizygotic twins. The heritability estimate was 60%, which confirms the importance of genetic factors in acute rheumatic fever. These findings provide a strong justification for embarking on whole genome mapping of genetic susceptibility variants for acute rheumatic fever in large appropriately designed cohorts. [1]

Twin studies provide the appropriate framework for assessing the extent to which the familial occurrence of acute rheumatic fever is due to genetic and environmental factors. Monozygotic and dizygotic twins generally share similar postnatal environments; hence phenotypic similarity in monozygotic twins that is greater than that of dizygotic twins is due to their greater genetic similarity. [27] To compare the different studies, we used the probandwise concordance rate to derive the proportion of affected co-twins for an affected proband in a particular study; the probandwise concordance rate has the advantage of providing the opportunity to compare studies irrespective of their ascertainment method, and provide an overall estimate of twin concordance rates for a particular condition. The important role which genetic susceptibility studies can play in informing the prioritization of research resources is illustrated in the unraveling of genes involved in breast cancer, where monozygotic twin pairs carry a risk of almost three times of developing the disease compared with dizygotic twins or first-degree relatives. [28]

Acute rheumatic fever is a complication of untreated streptococcal pharyngitis for which only affects 3–6% of the general population is at risk. It is not possible at present to predict the individuals who are at risk of developing acute rheumatic fever following an episode of streptococcal pharyngitis. The identification of genetic variants that reliably predict risk of development of acute rheumatic fever may be used to identify individuals who may benefit from prophylaxis with penicillin or vaccination against invasive streptococcal infection. This study suggests that genetic factors may have a high predictive power for the development of acute rheumatic fever, and as such may be of clinical utility in predicting disease risk. Therefore, the identification of all genetic susceptibility factors for acute rheumatic fever through whole genome analysis may lead to the development of a useful predictive genetic risk score for the disease.

This review has several limitations. First, there is the possibility of misclassification of the phenotype given that we used the clinical definition of acute rheumatic fever provided by the authors. Three of the studies were published before the original Jones criteria for the diagnosis of acute rheumatic fever were formulated in 1944, and relied on the clinical judgment of the investigators. Thus, given that the criteria for the diagnosis of acute rheumatic fever have evolved over the years, the generalizability of our findings to the present may be limited. Secondly, there was incomplete information on the age and gender of the twins included in this review. It is therefore not possible to assess the effects of age of onset or gender on genetic susceptibility to acute rheumatic fever in this work. Thirdly, there is a lack of data on the methods of determining zygosity status in four of the studies which may cast doubt on the reliability of the findings. Finally, it must be acknowledged that the results of twin studies cannot automatically be generalized beyond the population in which they were derived, and heritability is specific to a particular population in a particular environment. Furthermore, these studies were carried out over 45 years ago mainly in industrialized countries where endemic rheumatic fever has since been eradicated through improved living conditions and the availability of penicillin; today acute rheumatic fever is largely a disease of poor communities in developing countries. No studies of familial aggregation of the disease have been done within the developing country setting, which possibly reflects the relative neglect of this disease by the research community. [29] It is interesting, however, to note that the proportion of people at risk of development of acute rheumatic fever following untreated streptococcal pharyngitis remains the same in all ethnic groups of the world. [1] It is likely therefore, that there are no major differences in the magnitude of genetic determination of acute rheumatic fever in human populations.

In summary, this meta-analysis of 435 twin pairs with acute rheumatic fever shows that the monozygotic twin concordance substantially exceeds the dizygotic twin concordance, and thus implicates genetic factors as playing a significant role in the etiology of the condition. These results should provide renewed impetus to the establishment of large-scale studies to unravel the genetic architecture of acute rheumatic fever and rheumatic heart disease. Finding reliable and reproducible genetic susceptibility variants for acute rheumatic fever will not only assist in elucidating the pathophysiological mechanisms of the disease, but also assist in identifying individuals at risk of the condition in affected communities.

## Supporting Information

Appendix S1 Search strategy for twin studies of rheumatic fever and rheumatic heart disease.
(DOCX)
