# Supplementary material for: Genetic Susceptibility to Acute Rheumatic Fever: A Systematic Review and Meta-Analysis of Twin Studies
Source: PLoS One. 2011 Sep 30;6(9):e25326. doi: 10.1371/journal.pone.0025326 (PMC3184125; doi:10.1371/journal.pone.0025326)
Supplement: Appendix S1 — Search strategy for twin studies of rheumatic fever and rheumatic heart disease. (DOCX) [file pone.0025326.s001.docx]

**Appendix 1:**

**Search Strategy for twin studies of rheumatic fever and rheumatic heart disease**

**Search performed on Pubmed**

1  RHEUMATIC FEVER OR RHEUMATIC HEART

2  FAMIL* OR TWIN OR ADOPTION

3  #1 AND #2

**Search performed on EMBASE (**[**www.embase.com**](http://www.embase.com)**)**

1 'RHEUMATIC FEVER'/SYN OR 'RHEUMATIC HEART'

2 FAMIL* OR 'TWIN'/SYN OR 'ADOPTION'/SYN

3 #1 AND #2

4 #1 AND #2 AND [HUMANS]/LIM AND [EMBASE]/LIM
